# Data on yield and soil parameters of three diverse tilled long-term experimental sites in Austria (2018–2022)

**DOI:** 10.1038/s41597-025-05086-6

**Published:** 2025-05-20

**Authors:** Alexandra Tiefenbacher, Andreas Schaumberger, Hans-Peter Kaul, Emanuel Zillner, Andreas Hans, Andreas Surböck, Gabriele Gollner, Jürgen Friedel, Pia Euteneuer, Megan Asanza-Grabenbauer, Claudine Egger, Veronika Gaube, Johannes Peterseil, Taru Sandén, Theresa Strobl, Heide Spiegel

**Affiliations:** 1 Department for Soil Health and Plant Nutrition, Austrian Agency for Health and Food Safety (AGES), Vienna, Austria; 2 University of Natural Resources and Life Sciences, Vienna, Department of Sustainable Agricultural Systems, Institute of Organic Farming, Vienna, Austria; 3 Agricultural Research and Education Center (AREC) Raumberg-Gumpenstein, Irdning-Donnersbachtal, Styria, Austria; 4 University of Natural Resources and Life Sciences, Vienna, Department of Crop Sciences, Institute of Agronomy, Tulln, Austria; 5 University of Natural Resources and Life Sciences, Vienna, Department of Crop Sciences, Experimental Farm, Gross-Enzersdorf, Austria; 6 University of Natural Resources and Life Sciences, Vienna, Department of Economics and Social Sciences, Institute of Social Ecology, BOKU University, Vienna, Austria; 7 Ecosystem Research and Environmental Information Management, Umweltbundesamt GmbH, Vienna, Austria

**Keywords:** Environmental impact, Agroecology, Carbon cycle

## Abstract

The agroecological “Marchfeld” cluster assessed the impact of tillage on primary production (yield) and selected soil parameters at three sites (two conventionally and one organically managed) from 2018–2022. The data were uniformly compiled in a data set. The examined factors were no, minimum (5–8 cm), reduced (10–15 cm) and conventional (25–30 cm) tillage. All measured parameters were documented in a state-of-the-art quality control approach and stored in the data set. The long-term experimental (LTER) sites have been operating for a long time (from 6–34 years), so that our parameters show accumulated historical developments that influence the present. The data is available for (re)use by others (scientists, stakeholders, etc.) on Zenodo for meta-analyses, process modelling and other environmental studies.

## Background & Summary

Almost 40% of the world’s terrestrial surface area is managed as agricultural land^1^. Those fields yield 95% of the global food either directly or indirectly, underlining the essential role of agricultural soils for human nutrition^2^. The exponentially growing world population and the increasing problems associated with climate change will put pressures on future global food sovereignty^3,4^. Moreover, biomass for energy and material use is also being increasingly produced on the limited resource soil. This calls for managing agricultural land in a sustainable manner to maintain soil fertility and feedstock supply for future generations.

In terrestrial ecosystems, a wide range of ecological processes and patterns extend over long periods of time and large spatial scales^5^. Through its multifactorial approach to agriculture^6^, long-term ecological research (LTER) can provide insights into those chemical, physical and biological processes that become apparent only after years or even decades. In contrast, short-term experiments offer insights into how a system is controlled at a specific time and place by a set of factors (for instance: initial limiting factors and their interactions). Importantly, however, agricultural systems are the summation of multiple components operating at various time scales. The initial response curves of the entire system or single components do not automatically show the direction of the long-term changes, for instance changes in soil organic carbon stocks^5,7^. Thus, LTER provides systemic insights into biological, chemical, hydrological and biophysical processes under temporal dynamics. The scientific knowledge gained is crucial, especially when LTER is accompanied by a factorial approach such as gradient studies or specific treatments that manipulate specific factors and measure key processes^5,8^.

Historically, tillage was used for weed control and to prepare the soil for subsequent planting^9^. The type of soil cultivation significantly influences the soil biosphere. In contrast to tillage, no- tillage (the practice of direct seeding) can reduce soil erosion^10,11^, improve nutrient cycling^12^, enhance the water infiltration capacity of the soil and, by providing an adequate cover crop, reduce evaporation in semi-arid and arid climates^13–15^. At the same time, no- tillage practices can result in lower crop yields than conventional ploughing^16,17^. The impact of no-tillage as a climate mitigation approach remains uncertain^8,18^. Namely, the soil can become more anaerobic under reduced tillage practices, which in turn can promote the production of N_2_O^16,19^. Conventional tillage damages the aggregates of the soil surface^20^, making the soil prone to soil erosion^21^. Furthermore, tillage enhances the mineralization of soil organic carbon (SOC), reducing the SOC stocks^22^, although the literature on subsoil effects is still scarce^8^.

This paper describes selected years (2018–2022) in an agricultural data set containing data on a) agricultural management, b) primary production (crop yield), c) soil parameters (organic carbon, nitrogen concentration) collected from the three LTER experimental sites. The three LTER sites are combined in a linked group, the LTER “Marchfeld” Cluster of the Pannonian region in Austria, with diverse crop rotations and different tillage systems. Two of the LTER study sites (Gross-Enzersdorf and Fuchsenbigl) are conventionally managed, while the third one (Rutzendorf; MUBIL) is organically operated. The tillage treatments consist of a reduced (soil depth 10–15 cm) and a mouldboard ploughing (25–30 cm) system at all sites. On the conventionally managed study sites, further gradations of tillage, namely no till and minimum tillage (5–8 cm), are implemented. The soil parameters are available for various soil depths, usually in 10 cm (MUBIL: 15 cm) increments up to 30, 50 or 100 cm for MUBIL, GE and FB, respectively.

Over five years (2018–2022), substantial records were stored and archived in the data set “Cluster Marchfeld”. The data set is available at Zenodo^23^ (10.5281/zenodo.15212569).

## Methods

### Field study sites

**Fig. 1:**
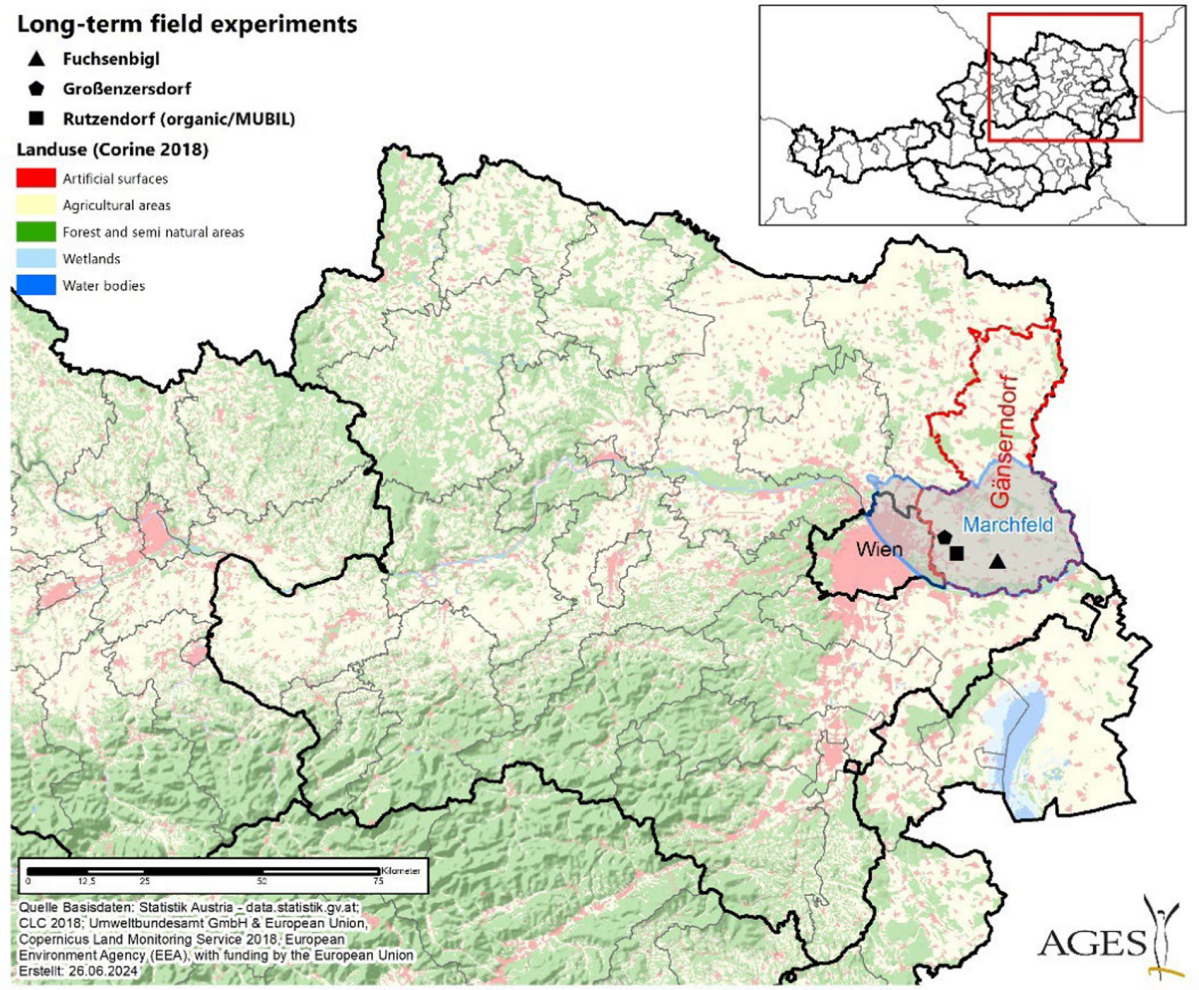
Location of field study sites within the Marchfeld region in Austria.

The three long-term experimental field sites are located in the Marchfeld region, which is part of the Pannonian basin, Austria (Fig. 1). Overall, the Marchfeld is one of the most important production areas for arable farming, including vegetables, in Central Europe^24^. This area is among the driest in Central Europe^25^ and is characterized by heightened wind erosion^26^, elevated nitrate concentrations in the groundwater^27^ and a limited amount of landscape elements^28^. A detailed description of the region “Marchfeld” is given in Guarini^29^. The socio- ecological trajectories are available at district level and the data were collected from the district “Gänserndorf”, because the Marchfeld region is part of that district. Furthermore, the three LTER sites have the most common soil types in the district Gänserndorf, i.e. Chernozem 50% and Phaeozem 12%^30^.

#### Fuchsenbigl (FB)

The tillage experiment, in Fuchsenbigl (FB, Marchfeld, AUSTRIA), was instigated in 1988 to evaluate the impact of tillage on soil physicochemical and biological properties as well as crop yields (Table 1). The following treatments were tested: A) **minimum tillage** with a rotary driller to a soil depth of 5–8 cm; B) **reduced tillage** with a cultivator to a soil depth of 15–20 cm; and C) **conventional tillage** with mouldboard ploughing to a depth of 25–30 cm^31^. The experiment consisted of experimental units with a plot size of 720 m^2^ (l × w: 60 m × 12 m). The experimental set-up is designed in a completely randomized block design with three replicates. On this study site, an open crop rotation is cultivated with the following most common crops: winter wheat (*Triticum aestivum* L.), soybean (*Glycine max* L.), winter barley (*Hordeum vulgare* L.) and winter triticale (*X Triticosecale* Wittmack), millet (*Sorghum bicolor* L.). A detailed description of the experiment design can be found in^31,32^.

**Table 1:** Overview of the three long-term experimental field sites.

	Fuchsenbigl (FB)	organic farm (MuBIL)	Gross-Enzersdorf (GE)
Literature reference	^43,44^	^45^	^46,47^
Operator	Austrian Agency for Health and Food Safety GmbH, Vienna	Institute of Organic Farming, University of Natural Resources and Life Sciences, Vienna	Institute of Crop Sciences, University of Natural Resources and Life Sciences, Vienna
GPS-Coordinates	48° 11′ N 16° 44′ E	48° 12′ N 16° 37′ E	48° 14′ N 16° 35′ E
Location	Fuchsenbigl, AUT	Rutzendorf, AUT	Groß-Enzersdorf, AUT
DEIMS ID	https://deims.org/09f126be-39db-4db8-af41-ac13cd12e8ea	https://deims.org/f3a6eebe-ae82-4fc6-a1ec-d9db723be139	https://deims.org/a5298d7f-307b-45d6-bd23-92ea7d65eed6
Year established	1988	2003 conversion to organic farming 2015 implementation of a tillage trial	1996
In 2022, the experiment ran for	34 years	19 years (fertilizer treatment) 6 years (tillage treatment)	26 years
Factor tillage (T) and cultivation depth (cm)	Minimum T: 5–8 Reduced T: 15–20 Conventional T: 25–30	Reduced T: 10–15 Conventional T: 25–30	No till Minimum T: 5–8 Reduced T: 8–10 Conventional T: 25–30
Soil type^33^	Haplic Chernozem	Calcearic Phaeozem	Calcaric Chernozem
MAT (mean annual air temperature)	11.2 °C (1991–2020)^48^		
MAP (mean annual precipitation)	560.4 mm (1991–2020)^48^		
Texture (% clay/silt/sand)	22/41/37	33/45/22	20/60/20
pH (CaCl_2_)	7.6	7.6	7.6
Soil Organic Carbon	1.20%	1.89%	2.33%

#### Organic farming Trial (“MUBIL”)

With its conversion to organic farming, the long-term field monitoring “MUBIL” (“Monitoring der Auswirkungen einer Umstellung auf den Biologischen Landbau”) was founded in 2003 (Table 1). The MUBIL trial is located in Rutzendorf (Marchfeld, AUSTRIA) and managed by the Institute of Organic Farming, University of Natural Resources and Life Sciences, Vienna (BOKU University). The soil is classified as a Calcaric Phaeozem^33^ with a soil pH_CaCl2_ of 7.6^34^ Table 1.

In 2003, an eight-year crop rotation was introduced at the MUBIL trial with the following sequence: 1^st^ Year: Lucerne (*Medicago sativa* L.) 2^nd^ Year: Lucerne; 3^rd^ Year: Winter wheat (*Triticum aestivum* L.) + catch crop; 4^th^ Year: Grain maize (*Zea mays* L.) 5^th^ Year: Spring barley *(Hordeum vulgare* L.*)* + catch crop; 6^th^ Year: Grain pea (*Pisum sativum* L.) + catch crop; 7^th^ Year: Winter wheat (*Triticum aestivum* L.) + catch crop; 8^th^ Year: Winter rye (*Secale cereale* L.*)*. The field plot trail was set up in a two-factorial, completely randomized block design with four replicates. The experiment consisted of experimental units with a plot size of 270 m² (15 m × 18 m). Four organic fertilization systems have been tested in the trial since 2003. In 2016, a new soil tillage trial was instigated at the MUBIL site in Rutzendorf. Prior to starting the experiment, the homogeneity of the soil from the whole site was examined and a medium-quality soil was selected for the experiment.

Two tillage treatments are tested in one organic fertilization system: A) one is ploughed with a **mouldboard plough** to a soil depth of 25–30 cm, while B) the other half is managed through **reduced tillage** by using a cultivator to a soil depth of 10–15 cm. All plots are fertilized by mulching Lucerne and/or catch crops.

#### Experimental farm gross- enzersdorf (GE)

The third long-term experimental field trail is located in Gross-Enzersdorf (Marchfeld, AUSTRIA, Table 1). In 1996, a soil tillage trial with the following treatments was instigated: A) **no- tillage** treatment: direct seeding in un-tilled soil with a disc drill without removing the previous crop residues. B) **minimal tillage** with a wing share cultivator to a soil depth of 8–10 cm; C) **reduced tillage** with a wing share cultivator (soil depth: 20–25 cm) and every four years the soil was tilled with a subsoiler to a depth of 35 cm; thus the crop residues remain only partly on the soil surface; and D) **mouldboard ploughing** to a depth of 25–30 cm, which implies incorporating the residues into the soil^35^.

The experiment was set up in a split plot design with four replicates. Thereby, the factor tillage was attributed to main plots (48 m × 40 m), whereas the factor crop rotation was assigned to subplots (24 × 40 m). The second factor, crop rotation, consisted of two levels: Treatment A) a four-year crop rotation with maize (*Zea mays* L.), winter wheat (*Triticum aestivum* L.), sugar beet (*Beta vulgaris* L.) and winter wheat (*Triticum aestivum* L.). And B) a four-year crop rotation with winter wheat (*Triticum aestivum* L.), soybean (*Glycine max* Merr.), winter wheat (*Triticum aestivum* L.), and oilseed rape (*Brassica napus* L.). The non-harvested crop residues remain on the field. In order to meet the nutritional requirements of main crops, the experimental site was fertilized according to good agricultural practices as indicated in the Austrian Guidelines. The whole experimental design is discussed in the following publications^35–37^:

### Agricultural management

All applied agricultural management practices were documented for 5 years, from 2018 to 2022. Mandatory data on management events were sowing (either main crop or cover crops), fertilization (type and quantity), harvest with crop name, tillage, mowing, integrated plant protection (type and quantity) and irrigation (if applied). Each activity and its associated device were described in detail. One aspect of it, the crop sequence of the three LTER sites for the years 2018–2022 is given in Table 2. The detailed list of agricultural management is given in the repository^23^ (10.5281/zenodo.15212569).

**Table 2:** Plan of crop cultivation (2018–2022).

Study sites	2018	2019	2020	2021	2022
FB	Sorghum	Winter wheat	Soy bean	Winter barley	Winter triticale
MUBIL	Grain Pea	Winter wheat	Winter rye	Lucerne	Lucerne
GE	Soy bean	Winter wheat	Soy bean	Winter wheat	Winter rapeseed

### Sampling

Grain yield and straw of the main crops were harvested and the dry matter content of the sampled materials was analysed. Every year, soil samples were taken annually in different soil depths. Soil samples were taken at the beginning of vegetation in spring (FB: end of February/beginning of March; MUBIL/GE: March/ April) and plant/crop samples were taken at the time of harvest.

### Analytical measurements

Twenty-three different analytical measurements were performed on all three LTER sites (Table 3). Some of those are project-specific (indicated by their year of analysis in the Table 3), but most of them have been applied on LTER sites since their year of origin. For our data set, we selected those years in which the chemical parameters were examined with the same analysis at the same accredited National reference laboratory in Austria (AGES). Thereby, the “total” and “systematic” measurement error variances are reduced. This is important, since both the analysis and performance of the laboratory could have an influence on the result itself^38^. Considering that, we have selected total organic carbon (TOC) using the dry combustion method^39^, total nitrogen using the^40^ and the C/N ratio of those analysis. Those soil parameters are available in various soil depths, usually in 10 cm (or 15 cm MUBIL) increments up to 30, 50 or 100 cm for MUBIL, GE and FB, respectively. The complete data set is available at Zenodo^23^ (10.5281/zenodo.15212569).

**Table 3:** All analyzed soil parameters.

Analysed parameter	Unit	Study sites		Method	Reference
		FB	MUBIL GE		
Total organic carbon (TOC)	%	X	18/21 2011	dry combustion	^39^
Total nitrogen (N)	%	X	18/21 2011	dry combustion	^40^
C/N ratio	—	X	18/21 2011	total C/total nitrogen	—

(This method was applied in all years: X, while some methods were applied in specific years: 2018 and 2021 (18/21) at MUBIL and 2020 at GE trial; N/A: Not available). Provided parameters are shown in bold.

## Data Records

The data set of the “ClusterMarchfeld” is online available in csv (Comma Separated Values) via Zenodo^23^ (10.5281/zenodo.15212569). The data set has been created using Microsoft Access 2019^41^. In total, the data set consists of 1153 records archived long- term data from 3 LTER sites. The data set consists of four csv Files: a) DataDescription (explains the headings of the csv files); b) Agricultural Management (offers Metadata to the experimental sites); c) Data Crop yield (provides yield data); d) Data Soil parameters (provides qualitative data on selected soil parameters).

The different sites are distinguished by their name (column: “Site_Name”: Fuchsenbigl; Gross-Enzersdorf; Rutzendorf), their farm management (column: “Farm_Category” either “organic” or “conventional” farming) as well as their respective crop rotation is shown (column: “Crop_Name”). The treatment tillage is given by the column “Tillage_Treatment” with the following gradations: “No till”; “Minimum tillage”; “Reduced tillage” or “Conventional tillage”. Soil parameters are available for various soil depths and it’s assigned soil layer (a range in cm; Column “Soil_Horizon”). The analyzed parameters are provided as “Crop_Yield_Dry”, “Total_Nitrogen”, “Total_Organic_carbon” or “C_N” ratio. Their corresponding units as well as their method of analysis are given through column “.._Unit” or “.._Method”, respectively (Table 4).

**Table 4:** Description of the provided data files at Zenodo^23^ (10.5281/zenodo.15212569), including column name, explanation as well as its range or group.

Column heading	Groups/ Range
**Site_Name**	Fuchsenbigl (FB); Gross-Enzersdorf (GE); Rutzendorf (MUBIL)
**Farm_Category**	Divides sites into “organic” or “conventional” operating fields
**Crop_Name**	Site-specific crop rotation at crop type level
**Tillage_Treatment**	Site-specific treatment with tillage following the gradients: No till (direct seeding); Minimum tillage; Reduced tillage or Conventional tillage
**Sampling_Date**	Date of sampling
**Soil_Horizon**	Displays the sampled soil horizon: Fuchsenbigl: 0–10; 10–20; 20–30; 30–40; 40–60; 60–80; 80–100; Gross-Enzersdorf: 0–5; 5–10; 10–15; 15–20; 20–25; 25–30; 30–40; 40–50; Rutzendorf: 0–15; 15–30;
**Crop_Yield; Total_Nitrogen; Total_Organic_Carbon; C_N**	Analyzed parameters at all three sites
**_Unit**	Corresponding unit of the analysed parameters
**_Method**	Corresponding method of analysis
**Management_Type**	Agricultural management is assigned to a type such as Seeding; Mowing; Harvest; Fertilization; Plant protection; Irrigation; Tillage
**Experimental_Unit**	Whether the agricultural management practice is applied on the whole (i.e seeding, harvest) or on the subplot (tillage). The tillage practice is further divided by the column Tillage_Treatment
**Management_Date**	Date of agricultural management
**Management_Description**	Further description of the applied agricultural management

The data of all applied agricultural management practices follows the following structure: column “Site_Name”, “Farm_Category” and “Management_Type” (Divided into the type of agricultural management such as Tillage, Seeding; Harvest, Plant Protection, Irrigation or Fertilization). General agricultural practices are applied to the entire trial, while the specific factors namely tillage are only applied to subplots. This is indicated with the column “Experimental_Unit” (either whole or subplot). Tillage practices are further divided into their assigned factor by the column “Tillage_Treatment”. All applied agricultural management practices are presented by the date (column “Management_Date”) and their description (column “Management_Description”). This data set is available at Zenodo^23^ (10.5281/zenodo.15212569).

## Technical Validation

The quality of the data is ensured on four levels:

A). **LTER study site**. Prior to starting the experiment, the homogeneity of fields was examined by measuring physical and chemical soil parameters. Compliance with the trial design, sample collection and all agricultural management measures were monitored and organized by the respective trial site managers. The collection of soil samples followed the guidance for sampling and storage^42^. The sampling campaign was conducted by a regularly trained sampling team.
B). **Laboratory**. The efficiency and effectiveness of the laboratory is regularly ensured by measuring reference materials, standard solutions, laboratory replicates, and by participating in interlaboratory comparisons. The maximum allowed relative standard deviation between replicates was set to 5%.
C). **Data collection**. In custom-made data templates, the data have been collected in an iterative manner. In those data templates, experiment names, treatment names, replicate number, observed year, measured value, units and methods are predefined to reduce the susceptibility to errors during data entry. Furthermore, each individual value was plotted to detect possible errors in the data input as well as to identify and correct deficiencies of the data input. An ANOVA was used to compare all measured parameters (crop yield as well as soil physical and chemical parameters) of each treatment. A pairwise comparison of treatments was performed with Tukey’s post-hoc tests (statistical significance set at p < 0.05). The completeness and quality of data was examined through data transfer templates, data verification checks, quality flagging and quality assessment exercises of the data set.
D). **Data set**. The presented data set has been publicly made available at Zenodo^23^ (10.5281/zenodo.15212569).

## Data Availability

No custom code was used.

## References

[1] Foley, J. A. *et al*. Solutions for a cultivated planet. *Nature* **478**, 337–342 (2011). 21993620 10.1038/nature10452

[2] Borrelli, P. *et al*. Land use and climate change impacts on global soil erosion by water (2015–2070). *Proc. Natl. Acad. Sci. USA.* **117**, 21994–22001 (2020). 32839306 10.1073/pnas.2001403117 PMC7486701

[3] Carvajal-Yepes, M. *et al*. A global surveillance system for crop diseases. *Science* **364**, 1237–1239 (2019). 31249049 10.1126/science.aaw1572

[4] Van Dijk, M., Morley, T., Rau, M. L. & Saghai, Y. A meta-analysis of projected global food demand and population at risk of hunger for the period 2010–2050. *Nat Food* **2**, 494–501 (2021). 37117684 10.1038/s43016-021-00322-9

[5] Knapp, A. K. *et al*. Past, Present, and Future Roles of Long-Term Experiments in the LTER Network. *BioScience* **62**, 377–389 (2012).

[6] Hobbie, J. E., Carpenter, S. R., Grimm, N. B., Gosz, J. R. & Seastedt, T. R. The US Long Term Ecological Research Program. *BioScience* **53**, 21 (2003).

[7] Gill, A. L. *et al*. Soil carbon availability decouples net nitrogen mineralization and net nitrification across United States Long Term Ecological Research sites. *Biogeochemistry* **162**, 13–24 (2023).

[8] Paz, A. M. *et al*. Collected knowledge on the impacts of agricultural soil management practices in Europe. *European J Soil Science* **75**, e13468 (2024).

[9] Reicosky, D. C. Conservation tillage is not conservation agriculture. *Journal of Soil and Water Conservation* **70**, 103A–108A (2015).

[10] Montgomery, D. R. Soil erosion and agricultural sustainability. *Proc. Natl. Acad. Sci. USA.* **104**, 13268–13272 (2007). 17686990 10.1073/pnas.0611508104 PMC1948917

[11] Nearing, M. A., Xie, Y., Liu, B. & Ye, Y. Natural and anthropogenic rates of soil erosion. *International Soil and Water Conservation Research* **5**, 77–84 (2017).

[12] Triplett, G. B. & Dick, W. A. No‐Tillage Crop Production: A Revolution in Agriculture! *Agronomy Journal* **100** (2008).

[13] Jones, A. *et al*. *The State of Soil in Europe: A Contribution of the JRC to the European Environment Agency’s Environment State and Outlook Report–SOER 2010*. (Publications Office, Luxembourg, 2012).

[14] Palm, C., Blanco-Canqui, H., DeClerck, F., Gatere, L. & Grace, P. Conservation agriculture and ecosystem services: An overview. *Agriculture, Ecosystems & Environment* **187**, 87–105 (2014).

[15] Pittelkow, C. M. *et al*. Productivity limits and potentials of the principles of conservation agriculture. *Nature* **517**, 365–368 (2015). 25337882 10.1038/nature13809

[16] Sanden, T. *et al*. European long-term field experiments: knowledge gained about alternative management practices. *Soil Use and Management* **34**, 167–176 (2018).

[17] Soane, B. D. *et al*. No-till in northern, western and south-western Europe: A review of problems and opportunities for crop production and the environment. *Soil and Tillage Research* **118**, 66–87 (2012).

[18] Ogle, S. M. *et al*. Climate and Soil Characteristics Determine Where No-Till Management Can Store Carbon in Soils and Mitigate Greenhouse Gas Emissions. *Sci Rep* **9**, 11665 (2019). 31406257 10.1038/s41598-019-47861-7 PMC6691111

[19] Powlson, D. S. *et al*. The potential to increase soil carbon stocks through reduced tillage or organic material additions in England and Wales: A case study. *Agriculture, Ecosystems & Environment* **146**, 23–33 (2012).

[20] Dignac, M.-F. *et al*. Increasing soil carbon storage: mechanisms, effects of agricultural practices and proxies. A review. *Agronomy for Sustainable Development* **37** (2017).

[21] Beniston, J. W. *et al*. Carbon and macronutrient losses during accelerated erosion under different tillage and residue management: Soil C, N and P losses during erosion. *European Journal of Soil Science* **66**, 218–225 (2015).

[22] Chenu, C. *et al*. Increasing organic stocks in agricultural soils: Knowledge gaps and potential innovations. *Soil and Tillage Research* **188**, 41–52 (2019).

[23] Tiefenbacher, A. *et al*. Cluster Marchfeld - Database on soil parameters and yields of diverse tillage systems from three LTER sites in Austria (2018–2022), *Zenodo*, 10.5281/zenodo.15212569 (2018).

[24] Raupp, J. *Long-Term Field Experiments in Organic Farming*. (Köster, Berlin, 2006).

[25] Hiebl, J., Reisenhofer, S., Auer, I., Böhm, R. & Schöner, W. Multi-methodical realisation of Austrian climate maps for 1971–2000. *Adv. Sci. Res.* **6**, 19–26 (2011).

[26] Scheper, S. *et al*. Comparison of the Spatial Wind Erosion Patterns of Erosion Risk Mapping and Quantitative Modeling in Eastern Austria. *Land* **10**, 974 (2021).

[27] Kasper, M. *et al*. N2O emissions and NO3− leaching from two contrasting regions in Austria and influence of soil, crops and climate: a modelling approach. *Nutr Cycl Agroecosyst* **113**, 95–111 (2019).

[28] Schirpke, U. *et al*. Past and future impacts of land-use changes on ecosystem services in Austria. *Journal of Environmental Management* **345**, 118728 (2023). 37536130 10.1016/j.jenvman.2023.118728

[29] Guarini, R., Bruzzone, L., Santoni, M. & Dini, L. Analysis on the effectiveness of multitemporal COSMO-SkyMed images for crop classification. in (ed. Bruzzone, L.) 964310, 10.1117/12.2193757 (Toulouse, France, 2015).

[30] NÖ Agrarbezirksbehörde. Soil MAP GÄNSERNDORF. *Unser Boden* http://www.unserboden.at/files/soilmap_gaenserndorf.pdf (2024).

[31] Spiegel, H., Dersch, G., Hösch, J. & Baumgarten, A. Tillage effects on soil organic carbon and nutrientavailability in a long-term field experiment in Austria. *Die Bodenkultur: Journal of Land Management, Food and Environment* 47–58 (2007).

[32] Hendricks, S. *et al*. Agricultural management affects active carbon and nitrogen mineralisation potential in soils. *J. Plant Nutr. Soil Sci.* **185**, 513–528 (2022).

[33] Working Group WRB, IUSS. *World Reference Base for Soil Resources 2014, Update 2015 Internation Soil Classifcation System for Naming Soils and Creating Legends for Soil Maps. World Soil Resources Reports No. 106. FAO, Rome*. (2015).

[34] Surböck, A., Heinzinger, M., Friedel, J. K. & Freyer, B. Monitoring der Umstellung auf den biologischen Landbau (MUBIL). (2006).

[35] Neugschwandtner, R. *et al*. Basic soil chemical properties after 15 years in a long-term tillage and crop rotation experiment. *Int. Agrophys.* **1**, 133–140 (2020).

[36] Liebhard, G., Klik, A., Neugschwandtner, R. W. & Nolz, R. Effects of tillage systems on soil water distribution, crop development, and evaporation and transpiration rates of soybean. *Agricultural Water Management* **269**, 107719 (2022).

[37] Moitzi, G., Neugschwandtner, R. W., Kaul, H.-P. & Wagentristl, H. Energy efficiency of winter wheat in a long-term tillage experiment under Pannonian climate conditions. *European Journal of Agronomy* **103**, 24–31 (2019).

[38] Burr, T., Kuhn, K., Tandon, L. & Tompkins, D. Measurement Performance Assessment of Analytical Chemistry Analysis Methods using Sample Exchange Data. *IJC* **3**, p40 (2011).

[39] ÖNORM L 1080. Soil and waste properties - Determination of organic carbon and humus content by dry combustion taking into account carbonates and elemental carbon. (2021).

[40] ÖNORM EN 16168. Sludge, treated biowaste and soil - Determination of total nitrogen using dry combustion method. (2012).

[41] Microsoft Corporation. Microsoft Access. (2019).

[42] ISO 10381-2:2002. Soil quality — Sampling — Part 2: Guidance on sampling techniques. (2002).

[43] Spiegel, H., Dersch, G., Hösch, J. & Baumgarten, A. Tillage effects on soil organic carbon and nutrient availability in a long-term field experiment in Austria. *Die Bodenkultur* (2007).

[44] Trajanov, A., Spiegel, H., Debeljak, M. & Sandén, T. Using data mining techniques to model primary productivity from international long-term ecological research (ILTER) agricultural experiments in Austria. *Regional Environmental Change* **19**, 325–337 (2019).

[45] Thomsen, I. K. *et al*. Management Effects on Quality of Organically Grown Winter Wheat. in vol. 37:2 172–192 (Agroecology and Sustainable Food Systems 2013).

[46] Euteneuer, P. *et al*. Cover crops affect soybean yield components, but not grain quality. *Agronomy Journal* **114**, 3193–3205 (2022).

[47] Neugschwandtner, R. W., Kaul, H.-P., Liebhard, P. & Wagentristl, H. Winter wheat yields in a long-term tillage experiment under Pannonian climate conditions. *Plant Soil Environ.* **61**, 145–150 (2015).

[48] ZAMG. Klimamittelwerte 1991–2020. https://www.zamg.ac.at/cms/de/klima/informationsportal-klimawandel/daten-download/copy_of_klimamittel (2024).

